# Gas Sensors Based on Electrospun Nanofibers

**DOI:** 10.3390/s90301609

**Published:** 2009-03-09

**Authors:** Bin Ding, Moran Wang, Jianyong Yu, Gang Sun

**Affiliations:** 1 State Key Laboratory for Modification of Chemical Fibers and Polymer Materials, College of Materials Science and Engineering, Donghua University, Shanghai 201620, P.R. China; E-Mail: binding@dhu.edu.cn (B.D.); 2 Nanomaterials Research Center, Modern Textile Institute, Donghua University, Shanghai 200051, P.R. China; E-Mail: yujy@dhu.edu.cn (J.Y.); 3 Los Alamos National Laboratory, Los Alamos, NM 87545, USA; E-Mail: mwang@lanl.gov (M.W.); 4 Fibers and Polymer Science, University of California, Davis, CA 95616, USA; E-mail: gysun@ucdavis.edu (G.S.)

**Keywords:** Gas sensors, electrospinning, nanofibers, acoustic wave, resistive, photoelectric, optical

## Abstract

Nanofibers fabricated via electrospinning have specific surface approximately one to two orders of the magnitude larger than flat films, making them excellent candidates for potential applications in sensors. This review is an attempt to give an overview on gas sensors using electrospun nanofibers comprising polyelectrolytes, conducting polymer composites, and semiconductors based on various sensing techniques such as acoustic wave, resistive, photoelectric, and optical techniques. The results of sensing experiments indicate that the nanofiber-based sensors showed much higher sensitivity and quicker responses to target gases, compared with sensors based on flat films.

## 1. Introduction

A gas sensor is a device which detects the presence of different gases in an area, especially those gases which might be harmful to humans or animals. The development of gas sensor technology has received considerable attention in recent years for monitoring environmental pollution. It is well-known that chemical gas sensor performance features such as sensitivity, selectivity, time response, stability, durability, reproducibility, and reversibility are largely influenced by the properties of the sensing materials used [1–3]. Many kinds of materials such as polymers [4,5], semiconductors [6,7], carbon graphites [8,9], and organic/inorganic composites [10,11] have been used as sensing materials to detect the targeted gases based on various sensing techniques and principles. It is worth noting that the sensitivity of chemical gas sensors is strongly affected by the specific surface of sensing materials [12,13]. A higher specific surface of a sensing material leads to a higher sensor sensitivity, therefore many techniques [14–16] have been adopted to increase the specific surface of sensing films with fine structures, especially to form the nanostructures, taking advantage of the large specific surface of nanostructured materials.

**Figure 1.**
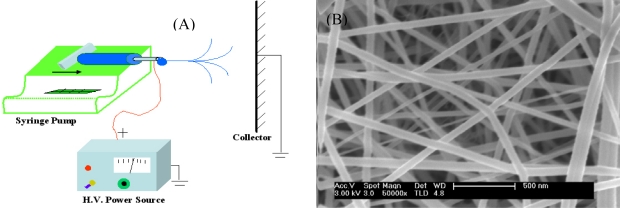
(A) A schematic of electrospinning process. (B) A SEM image of typical electrospun fibers.

Electrospinning is an efficient, relatively simple and low cost way to produce polymer and composite fibers with diameters ranging from several nanometers to a few micrometers by applying a high voltage to a polymer solution or melt ejected from a micro-syringe pump [17–19]. The ultrafine fibers produced via electrospinning are assembled as a three dimensional structured fibrous membrane with controllable pore structure and high specific surface area. A schematic of the electrospinning process and a typical SEM image of electrospun fibers are shown in Figure 1. Electrospinning is a special case of the electrospray process which uses an electrostatic field to form and accelerate liquid jets from the tip of a needle [20,21]. The surface of a hemispherical liquid drop suspended in equilibrium will be distorted into a conical shape in the presence of an external electric field. This distortion is caused by a balancing of the repulsive force resulting from the induced charge distribution on surface of drop with the surface tension of liquid [22]. A stable jet of liquid can be ejected and accelerated if the applied voltage exceeds this critical voltage. The jet breaks up into droplets as a result of the longitudinal Rayleigh instability caused by surface tension in the case of low viscosity liquids. This process is known as electrospray for applications requiring aerosols composed of sub-micron droplets with narrow distribution [23,24]. For high viscosity liquids, polymer solutions or melts, the jet does not break up, but travels as a jet to the grounded target. A transverse instability or splaying of the jet into two or more smaller jets is observed due to the radial charge repulsion [18]. This process is termed as “electrospinning” and it produces the polymer and composite fibers with diameters on the sub-micron scale [17].

To date, it is believed that more than one hundred different polymers have been successfully electrospun into nanofibers by this technique. Generally, the electrospun fibers are deposited on a fixed collector in a three-dimensional nonwoven membrane structure with a wide range of fiber diameter distribution. Extensive research on electrospinning show that the fiber collectability, uniformity of fibers, average fiber diameter, fiber diameter distribution, and fiber porosity are strongly affected by the solution properties [19,25] and processing parameters [26,27]. More recently, aligned electrospun fibers were obtained by using a rotating or pre-patterned collector [28,29]. Besides polymer and composite nanofibers, various inorganic semiconductor nanofibers are fabricated by sintering composite electrospun nanofibers in an elevated temperature [30–32]. The nanofibrous membranes with specific surface approximately 1–2 orders of the magnitude larger than the flat films have been targeted with applications in filtration [33], tissue engineering [34], drug delivery system [35], catalysis [36], dye-sensitized solar cells [37], battery separators [38], etc.

Electrospun fibers with controllable membrane thickness, fine structures, diversity of materials, and large specific surface are expected to be an ideal candidate as the structure of sensing materials. So far, many attempts (listed in Table 1) are carried out to prepare ultrasensitive gas sensors to detect vapors of NH_3_, H_2_S, CO, NO_2_, O_2_, CO_2_, moisture, and VOCs (CH_3_OH, C_2_H_5_OH, C_5_H_10_Cl_2_, C_6_H_5_CH_3_, C_4_H_8_O, CHCl_3_, C_2_H_2_Cl_2_, C_3_H_6_O, C_3_H_7_NO, C_2_HCl_3_, N_2_H_4_, (C_2_H_5_)_3_N, C_6_H_14_, etc.) with new and improved detection limits using electrospun nanofibrous membranes as sensing structures. The types of prepared gas sensors mainly include acoustic wave, resistive, photoelectric, and optical gas sensors. Electrospun fibers with polyelectrolyte components, conducting polymer composites, and semiconductors are successfully applied as gas sensing interfaces with the fiber arrangement of single fiber, oriented fibers, or nonwoven membranes at room or elevated operating temperature.

The fabrication and applications of nanofibers via electrospinning have been reviewed previously [39–42]. However, to the best of our knowledge, there have been no comprehensive reports on gas sensors based on electrospun nanofibers. In this review, we summarize the applications of electrospun fibers as gas sensing materials in recent years and call the attention of both scientists and engineers to this interesting research area.

## 2. Sensors

### 2.1. Acoustic Wave Gas Sensors

Quartz crystal microbalances (QCMs), one of a broad class of acoustic wave techniques, have been applied to detect small mass uptake on the order of nanograms when used for the characterization of thin layers, fluids, and gases due to their high mass sensitivity and durability [43]. According to Sauerbrey Equation (1) [44]:

(1) $$\Delta f=-f_{0}{}^{2}\Delta m/\text{NS}\rho$$

where Δf is the frequency change, f_0_ is the frequency of the quartz plate, Δm is the weight of applied coating, N is the unique constant of quartz crystal, S is the surface area of coated electrode, and ρ is the density of the quartz plate. QCM technology has been widely applied to the area of environmental monitoring, gas sensors, pressure sensors, and chemical sensors [45]. The sensitivity of QCM gas sensors is highly increased in the presence of a coating that interacts with the target gas [46].

**Figure 2.**
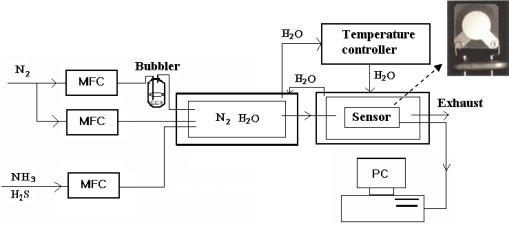
Schematic of a flow-type gas testing system for polyelectrolyte nanofiber gas sensors. (reproduced with permission from ref. [47]).

Ding *et al* [47] first applied electrospun nanofibrous membranes on QCM electrodes as highly sensitive gas sensors in 2004. A series of composite fibers of polyacrylic acid (PAA) and poly(vinyl alcohol) (PVA) with diameters of 100–400 nm containing various weight percentages of PAA to PVA were deposited on QCM via electrospinning and characterized with regards to their morphology and sensitivity to NH_3_. PAA, a weak anionic polyelectrolyte, interacts with NH_3_ gas. However, it is difficult to obtain nanofibers by electrospinning a pure PAA solution because of the formation of strong hydrogen bonds between water and the large amount of carboxyl groups in PAA. Therefore, the water-soluble PVA was introduced as a template to form composite fibers. Subsequently, heat treatment was employed to induce the crosslinking between PAA and PVA for reinforcing the fiber anti-water solubility. Sensing experiments were carried out at room temperature (RT) in a flow-type gas testing system (Figure 2) by measuring the resonance frequency shifts of QCM which due to the additional mass loading. The flow rates of dry N_2_, wet N_2_, and ammonia were kept constant by mass flow controllers (MFCs).

**Figure 3.**
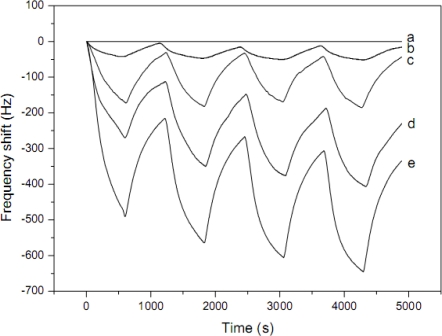
Response of nanofibers coated QCM sensors with various weight percentage of PAA to PVA exposed to 50 ppm of NH_3_ at the relative humidity of 55%. (a) 0 wt%; 11 wt%; 18 wt%; (d) 25 wt%; (e) 33 wt%. (reproduced with permission from ref. [47]).

The fiber sensors showed fast responses to NH_3_ after adding the PAA component in fibers (Figure 3). The average resonance frequency shifts of fibers coated QCM sensors with 11, 18, 25, and 33 wt% of PAA to PVA were 40, 150, 240, and 380 Hz, respectively. The sensing results indicated the content of PAA component, concentration of NH_3_, and relative humidity in gas testing chamber were all important parameters influencing the sensitivity of QCM sensors for NH_3_. Compared with the casted film-coated QCM sensors, the nanofiber-coated QCM sensors showed much higher sensitivity.

In order to further increase the fiber sensor sensitivity towards ammonia, microsized pure PAA fibers were deposited on the QCM electrodes with various solvent compositions of H_2_O and ethanol [48]. The connectivity between the electrode and fibrous membranes was designed to be enhanced by the incomplete evaporation of solvent in fibers with a short tip-to-electrode distance of 5 cm.

**Figure 4.**
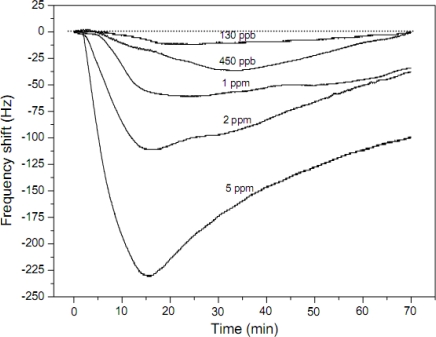
Response of the fibers coated QCM sensors with 10,000 Hz coating load exposed to various concentration of ammonia. The PAA fibrous membrane was obtained from the solvent of H_2_O and the relative humidity was 40% (reproduced with permission from ref. [48]).

The results of sensing experiments indicated that the sensitivity of the fibrous PAA membrane-coated QCM sensors was four times higher than that of the casted PAA film-coated QCM sensors. Moreover, the fiber-coated QCM sensors with small average fiber diameters had higher sensitivity towards low concentrations of ammonia, as low as 130 ppb at a relative humidity of 40 % (Figure 4).

However, the fiber QCM sensors could not detect low concentrations of ammonia gas at very low relative humidity. Therefore, the pre-sorbed moisture in fibrous membranes was proven to be the key factor affecting the sensitivity of QCM sensors for ammonia. Furthermore, the fiber-coated QCM sensors were found to have cross-sensitivity to ammonia and H_2_O, and the sensitivity of these sensors to moisture was much higher than to ammonia. Hence, they have a strong potential application in humidity sensors. Nanoporous films consisting of weak polyelectrolytes and TiO_2_ nanoparticles fabricated by a layer-by-layer (LBL) self-assembly method were applied on the electrode of QCM as ammonia gas sensors were prepared by Kim *et al* [49]. Compared to the sensing performance of nanoporous sensing films (60 Hz response to 10 ppm ammonia), the fiber-based gas sensors showed much higher sensitivity (230 Hz response to 5 ppm ammonia). However, the polyelectrolyte fibers are not as stable as the nanoporous hybrid LBL films in high humid atmospheres. Further design to enhance the insolubility of the fiber in water is needed.

Additionally, a novel H_2_S gas sensor [50] with a detection limit of 500 ppb was successfully fabricated by electrospinning deposition on QCM of nanofibrous polyethyleneimine (PEI)/PVA membranes with diameters of 100–600 nm as sensing materials. The morphology of sensing electrospun nanofibrous membranes was strongly influenced by the molar composition ratios of PEI to PVA. The sensor sensitivity was demonstrated to be affected by the morphology of sensing materials – it increases as the specific surface and porosity increase. Moreover, the ambient temperature and relative humidity were found to play an important role in the sensor sensitivity. The sensor sensitivity decreased with the conditions of high temperature and low relative humidity. The selectivity of fiber-based sensors was examined with H_2_S, CH_3_SH, NH_3_ and benzene. The sensors were insensitive to NH_3_ and benzene, but sensitive to H_2_S and CH_3_SH. The frequency shift of sensors response to 1 ppm of H_2_S, CH_3_SH, NH_3_ and benzene, is 40, 27, 1 and 0.3 Hz, respectively, thus relatively good selectivity of the fiber sensors towards the sulfide gases was found.

### 2.2. Resistive Gas Sensors

Various kinds of conducting polymers such as polyaniline (PANI), poly(diphenylamine) (PDPA), polypyrrole, polythiophene, etc. are currently used in various applications including metallic interconnects in circuits, chemical sensors, and electromagnetic radiation shielding [51]. The conductivity of such polymers results from the existence of charge carriers and from the ability of those charge carriers to move along the bonds of the polymer chains. These conducting polymers show chemical selectivity, which allows them to act as excellent materials for the immobilization of gas molecules, and exhibit highly reversible redox behavior with a distinguishable chemical memory.

In 2004, Craighead *et al.* [52] reported the fabrication of ammonia gas sensors via a scanned-tip electrospinning method using a single 10-camphorsulfonic acid (HCSA) doped PANI/poly(ethylene oxide) (PEO) nanofiber with diameter of 100–500 nm on gold electrodes. The well-defined single fiber geometry allowed for the characterization of the fiber material and the sensor response. The sensors showed a rapid and reversible resistance change upon exposure to NH_3_ gas at concentrations as low as 500 ppb through the protonation and deprotonation of PANI. The response time of nanofiber sensors was understood by considering the diffusion of ammonia into the fiber and the reaction of ammonia with doped PANI. The response times of sensors with various diameters corresponded to radius-dependent differences in the diffusion time of ammonia gas into the fibers.

**Figure 5.**
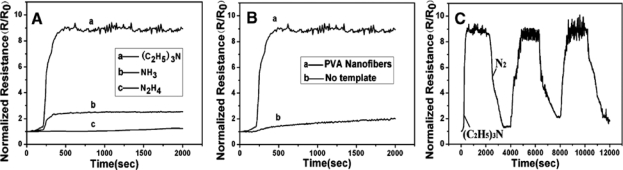
(A) Resistance change of PANI nanotubes exposed to 100 ppm of different gases (NH_3_, N_2_H_4_, and (C_2_H_5_)_3_N). (B) Response of PANI prepared by using PVA fiber mats as the template and without a template upon exposure to 100 ppm of (C_2_H_5_)_3_N. (C) The reversible circulation response change of PANI nanotubes upon exposure to 100 ppm of (C_2_H_5_)_3_N. (reproduced with permission from ref. [54]).

Another ammonia gas sensor with a detection limit of 1 ppm was prepared by Manesh *et al.* [53] using electrospun PDPA/poly(methyl methacrylate) (PMMA) nanofibers as sensing materials. The changes in resistance of the nonwoven membrane showed linearity with the concentration of ammonia in the range of 10–300 ppm. Furthermore, the detection target was expanded from ammonia to other amines (Figure 5) by Gong *et al* [54] using PANI nanotubes prepared using electrospun PVA fiber mats membrane as the template. The high surface areas, small diameter, and porous nature of the PANI nanotubes gave significantly better performance with regards to both sensitivity and time response. It was observed that the responses follow the orders: (C_2_H_5_)_3_N > NH_3_ > N_2_H_4_. Compared with PANI prepared without a template, the PANI nanotubes showed higher sensitivity and quicker response to (C_2_H_5_)_3_N. Additionally, the reversible circulation response change of PANI nanotubes has a reasonable reproducibility.

Li *et al* [55] obtained coaxial PANI/PMMA composite nanofibers using the electrospinning technique and an *in situ* polymerization method. The electrical responses of the gas sensors based on these composite nanofibers towards triethylamine (TEA) vapor were investigated at RT. It was revealed that the sensors showed a sensing magnitude as high as 77 towards TEA vapor of 500 ppm. In addition, the responses were linear, reversible and reproducible towards TEA vapor concentrations ranging from 20 to 500 ppm. Furthermore, it was found that the concentration of doping acids only led to changes in resistance of the sensor, but did not affect its sensing characteristics. The gas sensor with toluene sulfonic acid as the doping acid exhibited the highest sensing magnitude, which is explained by taking into account of the sensing mechanism and the interactions of doping acids with TEA vapor.

Other conducting composite electrospun fibers such as HCSA doped PANI [56], HCSA doped poly(*o*-toluidine) (POT)/polystyrene (PS) [57], and multi-walled carbon nanotubes (MWCNT) doped nylon [58] were fabricated as sensing materials to detect alcohols, moisture, hexane, acetone, ethyl acetate, dichloromethane, trichloromethane, tetrahydrofuran, and toluene vapors by measuring the resistance changes of fibers. However, there are no detailed concentrations of target vapors described in such reports. Therefore, the effects of temperature and vapor concentration should be investigated in the future work to fulfill the study of new sensing fibers. In addition, Tepper *et al.* [59] prepared electrospun fibers of carbon black (CB) doped polymers of poly(epichlorohydrin) (PECH), PEO, poly(isobutylene) (PIB), and poly(vinylpyrrolidone) (PVP) as sensing membranes to detect toluene, trichloroethylene, methanol, and dichloropentane vapors by measuring the electrical resistance of each element of the four-component sensor array. The sensor elements exhibited a linear response as a function of vapor concentration and response time ranged from several seconds to over 1 min.

Besides the conducting polymer composites, semiconducting metal oxides such as TiO_2_, SnO_2_, ZnO, WO_3_, and other wide band gap metal oxides are known for their ability to detect trace concentrations of various gases in air via charge transfer interactions between the sensor and chemisorbed species that modify the sensor’s resistance [60]. Kolmakov *et al.* [61] produced highly uniform SnO_2_ nanowires with bulk electronic properties directed by their surface chemistry through isolating and oxidizing tin nanowires selected from a template-synthesized array. The nanowires act as sensitive, fast, stable and reproducible gas sensors that can be easily integrated into a multi-component array. Recent efforts have been focused on the development of other nanostructured sensors to achieve increased surface-to-volume ratios and reduced cross sections, offering more effective gas modulation of device resistance.

**Figure 6.**
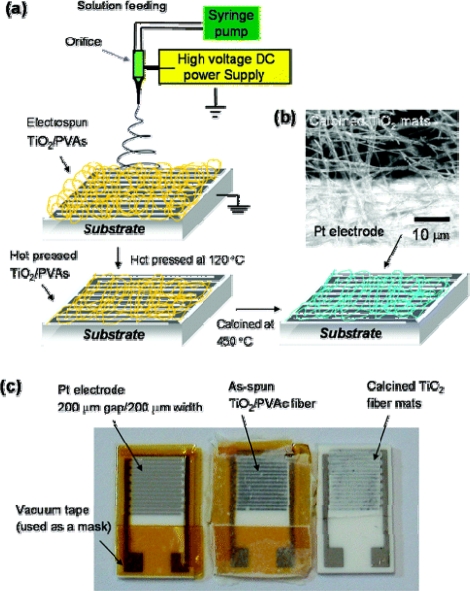
(a) Schematic diagram of the processing steps used to fabricate TiO_2_ nanofiber mats on Al_2_O_3_ substrates with interdigitated Pt electrode arrays. (b) Scanning confocal laser micrograph of a calcined TiO_2_ nanofiber mat on top of the Al_2_O_3_ substrate (dark region) and Pt electrode (bright region). (c) Optical micrographs of gas sensor test devices (10 mm × 15 mm) with TiO_2_ nanofiber mats after different processing steps. (reproduced with permission from ref. [62]).

**Figure 7.**
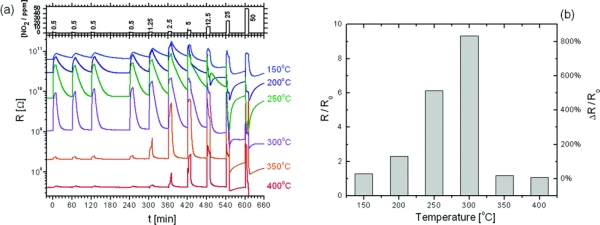
(a) The resistance response during cyclic exposure to 10 min pulses with increasing concentrations of NO_2_ mixed in dry air at various operating temperatures. (b) Sensitivity versus temperature histogram upon exposure to 500 ppb NO_2_ in dry air. *R*_0_ designates the baseline resistance in dry air. (reproduced with permission from ref. [62]).

An ultrasensitive chemiresistor based on electrospun TiO_2_ nanofibers [62] was fabricated by directly depositing TiO_2_/poly(vinyl acetate) (PVAc) composite nanofiber mats onto interdigitated Pt electrode arrays with subsequent hot pressing at 120 °C and calcining at 450 °C (Figure 6). The hot pressed TiO_2_ fibers had much higher specific surface area (138 m^2^/g) than that of unpressed fibers (31 m^2^/g) and led to a exceptionally high gas sensitivity. Exemplary results of the resistance response of one of the sensors upon cyclic exposure to NO_2_ at different concentrations and operating temperatures are shown in Figure 7. One can see that the resistance increased, for the most part, monotonically with NO_2_ concentrations of 500 ppb to 12.5 ppm and recovered reasonably fast, returning to the baseline resistance in dry air following each pulse of NO_2_-enriched air. The sensitivity versus temperature histogram went through a peak at 300 °C. The histogram’s shape resulted from the competition between slow kinetics at low temperatures and enhanced desorption at high temperatures. Unusual response patterns were observed at high NO_2_ concentrations (>12.5 ppm), consistent with n to p inversion of the surface-trap limited conduction facilitated by the high surface-to-volume ratio of this material.

A further study [63] on TiO_2_ nanofibers as sensing membrane to detect traces of CO and NO_2_ in air was reported to demonstrate the processing-microstructure-properties correlation of ultrasensitive gas sensors. The sensor was more sensitive at 300 °C than at 400 °C, similar to the previous report in [62], demonstrating exceptional sensitivity toward NO_2_ displaying a change of 405% in the sensor’s resistance on exposure to 50 ppb of NO_2_. By extrapolating the results to lower gas concentrations they estimated the detection limit to be 5 ppb for NO_2_ and 20 ppb for CO with operating temperature at 300 °C. Additionally, a highly sensitive and stable humidity nanosensors based on LiCl doped TiO_2_ electrospun nanofibers was reported by Wang *et al* [64]. The as-prepared humidity sensor exhibited excellent sensing characteristics, including ultrafast response time (≤ 3 s) and recovery time (≤7 s) for measuring relative humidity in a wide range of 11–95% in air at RT with the impendence changing from 107 to 104 Ω. Moreover, the nanosensor has good reproducibility, linearity, and stability.

Wang *et al* [65] reported the fabrication of a gas sensor to detect moisture and methanol gas based on a single SnO_2_ nanofiber made from C_22_H_44_O_4_Sn/PEO using electrospinning and metallorganic deposition techniques. The sensors showed high sensitivity to both gases and response times of the complete testing system were in the range of 108–150 s for moisture, and 10–38 s for methanol gas, respectively. Nonwoven type fibrous SnO_2_ gas sensors [66] for detection of ethanol were prepared on a micro hot plate by “near-field” electrospinning PVA/SnCl_4_ solution with a subsequent annealing at 300, 500, and 700 °C. The fiber resistance response increased with a raise of operating temperature and attained a maximum at 330 °C, followed by a decrease with further increases of operating temperature. The SnO_2_ nanofibers with an average diameter of similar to 100 nm exhibited large responses, low detection limits, fast response/recovery, and good reproducibility. The detection limit was <10 ppb and the response/recovery time towards 10 ppm ethanol was <14 s. Tao *et al.* [67] demonstrated a sensitive CO gas sensor for use at room temperature using polycrystalline SnO_2_ and MWCNT doped SnO_2_ nanofibers via electrospinning. The *n*-type MWCNT/SnO_2_ nanofibers are able to detect CO in the concentration range of 47–547 ppm.

The fabrication and characterization of polycrystalline WO_3_ nanofibers and their application for ammonia sensing were reported by Wang *et al.* [68]. Pure tungsten oxide nanofibers with controllable diameters of around 100 nm were obtained by electrospinning PVAc/tungsten isopropoxide followed by a calcinations step. The relationship between solution concentration and ceramic nanofiber morphology has been studied. It has been shown that the as-prepared tungsten oxide ceramic nanofibers have a quick response to ammonia with various concentrations at 350 °C, suggesting potential applications of the electrospun tungsten oxide nanofibers as a sensor material for gas detection. Sahner *et al* [69] fabricated a perovskite *p*-type conducting electrospun SrTi_0.8_Fe_0.2_O_3-δ_ nanofibrous membrane as a model sensing material for application in methanol sensing at the operating temperature of 400 °C. When exposed to reducing gases, the sensor resistance increased, as expected for a *p*-type sensor membrane. Very good sensor characteristics such as short response and recovery times, and a stable baseline can be observed. At 10 ppm methanol, sensor response *R*/*R_0_* was observed to be 5.5 (electrospun) and 2.9 (electrosprayed). In the case of the electrospun membrane, the signal for methanol with concentrations ranging from 5 to 50 ppm in dry air was found to be reproducible.

### 2.3. Photoelectric Gas Sensors

Most gas sensors based on one dimensional nanosized ZnO have to be operated at an elevated temperature in order to exhibit their sensing behavior, which is significantly inconvenient for practical applications. Xie *et al.* [70] thought to use light excitation instead of increasing temperature to change conductance owing to the distinguished optical and electric performance of ZnO. To extend the photoelectric response of wide-band gap semicondutors into the visible spectral range and utilize solar energy effectively, nonwoven type Co doped ZnO nanofibers with diameter of 50–400 nm were deposited on ITO comb-like electrodes by electrospinning PVP/zinc acetate/cobalt acetate solution with a subsequent annealing at 500 °C for 3 h. The surface photocurrent (*I*_SPC_) of electrospun Co-ZnO fibers was measured with the comb-like electrodes using the light source-monochromator-lock-in detection technique. To exploit the application of Co doped ZnO fibers for a photoelectric sensor, four values of oxygen pressure: 9.28, 4.25, 4.06, and 1.16 Torr were used. The sensors showed a very short response and recovery time to oxygen, as well as the good reproducibility under the illumination of a Xe lamp. The response time, corresponding to 70% of the equilibrium value, was about 1 min. Meanwhile, the change of *I*_SPC_ was linear to the oxygen concentrations with a detection limit of 0.322 Torr.

### 2.4. Optical Gas Sensors

Optical fiber sensors have many advantages compared to traditional types of sensors such as the absence of the electromagnetic interference in sensing and electric contacts in the probe, multiplexity on a single net work, etc. Fourier transform infrared (FTIR) spectroscopy is one of the widely used optical methods to study the interaction of electromagnetic radiation in the infrared region with chemical compounds. The large band width (400–4500 cm^−1^) and the distinct absorbance bands render the FTIR technology suitable for sensor applications. Hahn *et al.* [71] first utilized electrospun nanocomposite fiber mats as optical sensors in conjunction with FTIR spectroscopy to detect CO_2_ gas. The nanocomposite fiber mats were prepared by electrospinning polyacrylonitrile (PAN) solutions containing nanoparticles including iron oxide, antimony tin oxide and zinc oxide with diameters ranging from 10 to 70 nm. The porosity increased from 70% for PAN fiber mat to an average of 86% for nanocomposite fiber mats. The absorbance spectra showed a higher sensitivity with a fiber mat, regardless of its type, than without, indicating gas adsorption on the fiber mat. The highest sensitivity was obtained with PAN/Fe_2_O_3_ fiber mat. As the underlying mechanism is physisorption rather than chemisorption, the response time was short and the sensor could be used repeatedly.

## 3. Conclusions

The utilization of electrospun nanofibers with large specific surface as gas sensing materials has received great attention since 2004. Various gas sensors comprising electrospun nanofibers of polyelectrolytes, conducting polymer composites, and semiconducting metal oxides were successfully fabricated with ultrahigh sensitivity, very short response and recovery time, and good reversibility, reproducibility, and stability based on various sensing techniques and principles. The fine structure of electrospun fibers was proven to be an excellent candidate instead of the current widely used solid flat films to further increase the sensor sensitivity. It opens a new way to fabricate the ultrasensitive sensors. It can be foreseen that in the near future more and more ultrasensitive and practical electrospun nanofiber sensors will be fabricated based on the above reviewed publications. Furthermore, we suggest studying the specific surface area of fibrous membranes and the connectivity between the electrodes and nanofibrous membranes since they would have the large effects on sensing performance.

## Figures and Tables

**Table 1.** Types of electrospun nanofibers based gas sensors.

**Types**	**Materials**	**Array of fibers**	**Fiber diameter**	**Gases tested**	**Operating temperature (°C)**	**Detection limit**	**Ref.**

Acoustic wave	PAA-PVA	Nonwoven	100–400 nm	NH_3_	RT	50 ppm	[47]
	PAA	Nonwoven	1–7 μm	NH_3_	RT	130 ppb	[48]
	PEI-PVA	Nonwoven	100–600 nm	H_2_S	RT	500 ppb	[50]

Resistive	HCSA-PANI/PEO	Single	100–500 nm	NH_3_	RT	500 ppb	[52]
	PDPA-PMMA	Nonwoven	∼400 nm	NH_3_	RT	1 ppm	[53]
	PANI	Nonwoven	0.3–1.5 μm	Amines	RT	100 ppm	[54]
	PMMA-PANI	Nonwoven	250–600 nm	(C_2_H_5_)_3_N	RT	20 ppm	[55]
	HCSA-PANI	Single	20–150 nm	Alcohols	RT	No data	[56]
	HCSA-POT/PS	Nonwoven	0.2–1.9 μm	H_2_O	RT	No data	[57]
	MWCNT/nylon	Nonwoven	110–140 nm	VOCs	RT	No data	[58]
	CB-PECH,	Oriented	∼3 μm	CH_3_OH	RT	1000 ppm	[59]
	PEO, PIB, PVP			C_5_H_10_Cl_2_		5 ppm	
				C_6_H_5_CH_3_		250 ppm	
				C_2_HCl_3_		500 ppm	
	TiO_2_	Nonwoven	200–500 nm	NO_2_	150–400 °C	500 ppb	[62]
	TiO_2_	Nonwoven	120–850 nm	CO, NO_2_	300–400 °C	50 ppb	[63]
	LiCl-TiO_2_	Nonwoven	150–260 nm	H_2_O	RT	11%	[64]
	SnO_2_	Single	700 nm	H_2_O	RT	No data	[65]
	SnO_2_	Nonwoven	∼100 nm	C_2_H_5_OH	330 °C	10 ppb	[66]
	MWCNT/SnO_2_	Nonwoven	300–800 nm	CO	RT	47 ppm	[67]
	WO_3_	Nonwoven	20–140 nm	NH_3_	350 °C	50 ppm	[68]
	SrTi_0.8_Fe_0.2_O_3−δ_	Nonwoven	∼100 nm	CH_3_OH	400 °C	5 ppm	[69]

Photoelectric	Co-ZnO	Nonwoven	50–400 nm	O_2_	RT	0.32 Torr	[70]

Optical	Oxides-PAN	Nonwoven	50–200 nm	CO_2_	RT	700 ppm	[71]

Abbreviations in Table 1: PAA (polyacrylic acid); PVA (polyvinyl alcohol); PEI (polyethyleneimine); HCSA (10-camphorsulfonic acid); PANI (polyaniline); PEO (polyethylene oxide); PDPA (polydiphenylamine); PMMA (polymethyl methacrylate); POT (poly*o*-toluidine); PS (polystyrene); MWCNT (multi-walled carbon nano-tube); CB (carbon black); PECH (polyepichlorohydrin); PIB (polyisobutylene); PVP (polyvinylpyrrolidone); PAN (polyacrylonitrile); VOC (volatile organic compound); RT (room temperature).
